# A Multivariate Model to Predict Chronic Heart Failure after Acute ST-Segment Elevation Myocardial Infarction: Preliminary Study

**DOI:** 10.3390/diagnostics11101925

**Published:** 2021-10-18

**Authors:** Valentin Elievich Oleynikov, Elena Vladimirovna Averyanova, Anastasia Aleksandrovna Oreshkina, Nadezhda Valerievna Burko, Yulia Andreevna Barmenkova, Alena Vladimirovna Golubeva, Vera Aleksandrovna Galimskaya

**Affiliations:** Department of Therapy, Medical Institute, Penza State University, 440026 Penza, Russia; v.oleynikof@gmail.com (V.E.O.); averyanova-elena90@bk.ru (E.V.A.); anast.oreschckina@yandex.ru (A.A.O.); yulenka.gsk@gmail.com (Y.A.B.); fialmy@mail.ru (A.V.G.); vera-budanova@mail.ru (V.A.G.)

**Keywords:** myocardial infarction, chronic heart failure, heart rate variability, 2D echocardiography, 24-h ECG monitoring

## Abstract

A multivariate model for predicting the risk of decompensated chronic heart failure (CHF) within 48 weeks after ST-segment elevation myocardial infarction (STEMI) has been developed and tested. Methods. The study included 173 patients with acute STEMI aged 51.4 (95% confidence interval (CI): 42–61) years. Two-dimensional (2D) speckle-tracking echocardiography (STE) has been performed on the 7th–9th days, and at the 12th, 24th, and 48th weeks after the index event with the analysis of volumetric parameters and values for global longitudinal strain (GLS), global circumferential strain (GCS), and global radial strain (GRS). A 24-h ECG monitoring (24 h ECG) of the electrocardiogram (ECG) to assess heart rate turbulence (HRT) has been performed on the 7th–9th days of STEMI. The study involved two stages of implementation. At the first stage, a multivariate model to assess the risk of CHF progression within 48 weeks after STEMI has been built on the basis of examination and follow-up data for 113 patients (group M). At the second stage, the performance of the model has been assessed based on a 48-week follow-up of 60 patients (group T). Results. A multivariate regression model for CHF progression in STEMI patients has been created based on the results of the first stage. It included the following parameters: HRT, left ventricular (LV) end-systolic dimension (ESD), and GLS. The contribution of each factor for the relative risk (RR) of decompensated CHF has been found: 3.92 (95% CI: 1.66–9.25) (*p* = 0.0018) for HRT; 1.04 (95% CI: 1.015–1.07) (*p* = 0.0027) for ESD; 0.9 (95% CI: 0.815–0.98) (*p* = 0.028) for GLS. The diagnostic efficiency of the proposed model has been evaluated at the second stage. It appeared to have a high specificity of 83.3%, a sensitivity of 95.8%, and a diagnostic accuracy of 93.3%. Conclusion. The developed model for predicting CHF progression within 48 weeks after STEMI has a high diagnostic efficiency and can be used in early stages of myocardial infarction to stratify the risk of patients.

## 1. Introduction

Chronic heart failure (CHF) has become a major public health problem of the 21st century, significantly reducing the life potential of the population worldwide. Major causes of CHF are arterial hypertension and coronary heart disease [1,2]. A combination of both diseases occurs in more than half of patients [2,3].

Annual costs of treating CHF patients grow progressively due to an increase in the life expectancy of patients [4,5]. According to a large-scale meta-analysis [5], a five-year life expectancy in patients with CHF has increased by 59.7% as compared to the 1970s. Improvement of the quality of care and expanding the range of drugs after severe cardiovascular events help to maintain the quality of life of patients at an acceptable level for a long period of time. However, the financial aspect of this issue is rather burdensome [4,5,6].

Nowadays, patients with acute ST-segment elevation myocardial infarction (STEMI) receive high-quality treatment due to the achievements of interventional cardiology and drugs that are able to restore and “revive” hibernating cardiomyocytes in the infarct zone. Thus, the prognosis of patients after myocardial infarction has significantly improved in the era of pharmacoinvasive therapy [7,8]. According to the French Registry of Acute ST-Elevation or Non-ST-Elevation Myocardial Infarction [7], a six-month mortality rate in patients after STEMI decreased from 17.2% in 1995 to 6.9% in 2010, and 5.3% in 2015.

Due to the increase in the survival rate of patients with myocardial infarction, the prediction and prevention of CHF decompensation are the key tasks for the cardiological community [9].

Currently, there are some urgent problems, dealing with the introduction of the possibilities of laboratory and instrumental diagnostic methods to predict the risk of developing CHF decompensation, and how to reduce the risk of re-hospitalization of patients. To predict a high risk of CHF in early stages of STEMI is essential for personalized anti-remodeling therapy, reliable recommendations for therapeutic rehabilitation, and timely cardiac surgery.

The aim of this study was to create a model for reliable risk prediction of CHF progression after acute STEMI during a 48-week follow-up using a combination of echocardiography (EchoCG) and 24 h ECG monitoring.

## 2. Materials and Methods

A total of 1256 STEMI patients were examined in the Emergency Cardiology Department of the Regional Clinical Hospital n. a. N.N. Burdenko (Penza, Russia). According to evaluated inclusion/exclusion criteria, a total of 173 STEMI patients were enrolled in the study. The study protocol and informed consent were approved by the Local Ethics Committee at Penza State University.

The study included patients having met the following criteria: aged 35–65 years; acute STEMI of any localization, confirmed by laboratory and instrumental methods (increased troponin levels, ECG data, coronary angiography (CA), EchoCG). The main exclusion criteria were as follows: repeated and recurrent myocardial infarction; stenosis of more than 30% of the left coronary artery; stenosis of more than 50% of other coronary arteries, except for an infarct-related one; NYHA class II-IV of CHF; non-sinus rhythm; severe concomitant diseases.

The average age of the patients was 51.4 (95% CI: 42–61) years, men prevailed—152 patients (87.8%). Two-dimensional EchoCG has been performed on the 7th–9th days, and at the 12th, 24th, and 48th weeks after STEMI using a MyLab90 ultrasound scanner (Esaote, Genoa, Italy) with analysis of generally accepted volumetric parameters. The ejection fraction (EF) has been calculated using a modified Simpson method. The end-diastolic volume index (EDVI) and the end-systolic volume index (ESVI) have been determined by indexing to body surface area (BSA). Speckle-tracking EchoCG has been performed using Esaote XStrain™ software. The values for global longitudinal strain (GLS), global circumferential strain (GCS), and global radial strain (GRS) have been estimated [10].

A 12-lead 24-h ECG monitoring has been carried out using Holter Analysis-Astrocard complex devices (Meditek Ltd., Moscow, Russia) on the 7th–9th days of STEMI. Episodes of ischemia, arrhythmias have been assessed: ventricular and supraventricular extrasystoles, running of stable and unstable tachycardias, paroxysms of atrial fibrillation and flutter, ventricular fibrillation, conduction disturbances—sinoatrial and atrioventricular blockades. An analysis of heart rate turbulence (HRT) has been performed on the basis of the 24-h ECG. The deviation of at least one of two parameters has been taken as pathological HRT: turbulence onset (TO) <0%), and turbulence slope (TS) >2.5 ms/RR). Heart rate variability (HRV) has been assessed by temporal (SDNN—standard deviation of the average values of RR-intervals; SDNNi—mean value of standard deviations of RR intervals for a 5-min recording; SDANN—standard deviation of the average values of sinus RR intervals for 5 min), and spectral characteristics (TotP—total spectrum power; LfP—low-frequency component of the spectrum; HfP—high-frequency component of the spectrum; L/H—vagosympathetic balance index) [11].

The level of brain natriuretic peptide (BNP) has been determined on the 7th–9th days after STEMI. A six-minute walk test has been performed to determine NYHA class of CHF starting from the 12th week of the postinfarction period.

The progression of CHF in the postinfarction period has been considered to be the endpoint, determined by the development of one of the following events [12,13]: hospitalization of the patient due to decompensation of CHF; decrease in EF compared to baseline values with the patient’s transition from the group with preserved EF (HFpEF) to the group with mid-range (HFmrEF) or reduced (HFrEF) ejection fraction, or from HFmrEF to HFrEF group; a six-minute walk test results corresponding to NYHA class II-IV of CHF.

The STEMI patients have been receiving treatment in accordance with the ESC Clinical Practice Guidelines [14]. Percutaneous coronary intervention (PCI) with stenting (100%) has been carried out in all patients on the first day of STEMI. Twenty (33.3%) patients have undergone pharmacoinvasive reperfusion—systemic thrombolytic therapy (TT) and PCI.

The study involved two stages of implementation. At the first stage, a multivariate model for assessing the risk of CHF progression within 48 weeks after STEMI has been built on the basis of examination and follow-up data for 113 patients (group “M”). At the second stage, the sensitivity and specificity of the multivariate model for the risk of CHF decompensation has been tested based on a 48-week follow-up of 60 patients (group “T”).

Statistical data processing has been performed using the licensed version of STATISTICA 13.0 program (StatSoft, Inc., OC, OK, USA). All values of quantitative traits are given with the 95% confidence interval (CI). The McNemar criterion has been used when comparing qualitative characteristics for paired samples, and χ^2^ test has been used for independent samples. To determine the influence of the parameters on the endpoint development, and to estimate the relative risk (RR) and 95% CI, the method of univariate analysis by applying logistic regression has been used. The value of *p* < 0.05 was taken as a threshold of statistical significance. To include the indicators in a multivariate model using the Cox multiple linear regression, the absence of a correlation between thereof should be a prerequisite. To assess the information content and adequacy of the logistic model, the coefficients of sensitivity and specificity have been determined. Sensitivity (Se) is the ability of a diagnostic method to give a reliable result, and specificity (Sp) is the ability of a diagnostic method not to provide false positive results in the absence of a disease [15].

## 3. Results

According to the results of the first stage of the study [16], the endpoints had been achieved in 26 (23%) patients of group “M”. Nine (35%) patients were hospitalized due to decompensation of CHF; NYHA class III of CHF was estimated in 2 (7.7%) patients according to a six-minute walk test; a decrease in EF was determined in 19 (73%) patients with the transition from HFpEF: to HFmrEF—in 11 (58%), to HFrEF—in 4 (21%), from HFmrEF to HFrEF—in 4 (21%) patients.

The following factors for CHF progression have been established according to the univariate regression analysis of data from 113 STEMI patients: pathological values of TO and HRT, BNP level, ESD, GLS, GCS, and GRS values obtained on the 7th–9th days of STEMI (Table 1). The EF has shown no diagnostic significance in detecting CHF progression.

Considering correlations between given parameters and using a stepwise variable selection method, a multivariate regression model for CHF progression in STEMI patients has been created. It includes HRT, ESD, and GLS values, and has the form of the formula:

(1) $$h=h_{0}\left(t\right)\cdot \exp\left(1.366539\cdot X_{1}+0.043323\cdot X_{2}-0.108260\cdot X_{3}\right)$$

where: *X*_1_ is equal to 1.0 with pathological HRT on the 7th–9th days of STEMI, and it is equal to 0 with normal HRT; *X*_2_—ESD, mm; *X*_3_—GLS, %; *h*_0_(*t*)—baseline risk of 0.018611 at the 6th week, 0.100065 at the 12th week, 0.108673 at the 16th week, 0.181305 at the 24th week, and 0.212152 at the 48th week. The risk of predicting CHF has been calculated at the 6th, 12th, 16th, 24th, and 48th weeks of the postinfarction period. We have concluded that if the value of h was higher than 1.0, CHF progression was predicted; if the value of h was less than 1.0, there was a stable course of the postinfarction period.

The analysis of variance (ANOVA) has shown that the present model of differentiation of patients by the nature of the postinfarction period has a high information content: the Wilk’s Lambda = 0.83243, F (3109) = 7.3139 (*p* = 0.00016).

At the second stage, the performance of the developed multivariate model has been tested in group “T” (60 patients). Table 2 shows comparative characteristics of groups “M” and “T”. There were no significant differences in age, a number of concomitant conditions, and localization of the infarction area in the groups. However, the patients with burdened heredity for cardiovascular diseases were found more frequently in group “T”.

CHF progression was recorded in 12 (20%) patients of group “T” over the 48-week follow-up. One (8.3%) patient was hospitalized due to CHF decompensation; NYHA class III of CHF was estimated in 2 (16.7%) patients according to the six-minute walk test; a decrease in EF was determined in 9 (75%) patients: with the transition from HFpEF to HFmrEF—in 3 (25%), and from HFmrEF to HFrEF—in 6 (50%) patients.

Sensitivity, specificity, and diagnostic accuracy of the method have been determined to assess the adequacy of the presented logistic regression individual risk model for predicting CHF.

Table 3 shows the results of testing a multivariate model for predicting CHF progression in patients of group “T” (*n* = 60) within 48 weeks after STEMI.

The diagnostic model is characterized by low sensitivity with a high level of specificity and diagnostic accuracy at the 12th week. However, sensitivity of the model has increased to 83.3% by the 48th week at the later stages of the postinfarction period. Thus, the individual risk model for predicting CHF after STEMI has acceptable sensitivity, high specificity, and diagnostic accuracy and can be used in early stages of STEMI (on the 7th–9th days) to identify patients with a high risk of CHF decompensation.

## 4. Discussion

Late and/or unsuccessful pharmacoinvasive revascularization, large-size myocardial infarction, life-threatening arrhythmias, old and senile age, lack of previous drug therapy of cardiovascular diseases, type 2 diabetes, and comorbidity are known as factors to be associated with a high risk of CHF progression [4,5,12,17]. Various combinations thereof affect the risk of developing CHF in different ways, which, however, requires proof in appropriate multivariate models.

BNP is one of the most common CHF indicators [12,18]. According to some researches [19], such biomarkers as N-terminal pro-brain natriuretic peptide (NT-proBNP), pentraxin-related protein (PTX-3) and, to a lesser extent, stimulating growth factor 2 (ST2), have demonstrated their prognostic significance in diagnosis of cardiovascular complications with a sensitivity of 78.79% and a specificity of 86.67% (area under the curve, AUC 0.73). The current study has also established the role of BNP in long-term prognosis in STEMI patients, but this parameter has not been included in the multivariate model.

Investigation of cardiac biomechanics is essential in assessing the function of the entire cardiovascular system in patients with myocardial infarction, since this group of patients has a high risk of CHF progression due to left ventricular (LV) pathological remodeling. As LV EF is the most studied, the expediency of its monitoring in CHF is beyond doubt, since reduced ejection fraction is associated with an unsatisfactory prognosis, poor quality of life of patients, increased mortality and hospitalization rates [4,5,12,20]. A decrease in LV EF during dynamic follow-up is a direct indicator of CHF progression. Despite that a decrease in EF with the transition of a patient from HFpEF to HFmrEF or HFrEF, and from HFmrEF to HFrEF in the post-infarction period, has been regarded as one of the manifestations of CHF progression in the current study, the initial values of the LV EF on the 7th–9th days of STEMI did not demonstrate diagnostic significance in detecting CHF progression in the postinfarction period.

Many researchers have found that HRV is a valid factor for a high risk of mortality and CHF progression [11,21]. Low values of parameters for HRV spectral analysis (HfP) and temporal analysis (SDAAN) are associated with an increase in the probability of CHF decompensation.

HRT is an appropriate technique for stratification of CHF risk in postinfarction patients [11]. A group of researchers led by Cygankiewicz [22] have established a correlation between HRT, EF, and class of CHF. In the current study, it has been revealed that pathological values of TO and impaired HRT on the 7th–9th days of STEMI were independent factors of CHF decompensation.

Another urgent problem is the risk stratification of life-threatening rhythm disturbances in CHF patients who have had myocardial infarction. Due to the lack of an accurate system for determining the risk of sudden death in such patients, implantation of a cardioverter defibrillator is not always properly performed [23,24,25]. Some researchers believe that a deeper investigation of the global strain characteristics would help unify the selection of patients [26,27]. Special attention is paid to the GLS parameter, since it has been established to be an indicator of an unfavorable course of CHF, and is associated with a decrease in the functional status of patients and an increase in the volume of the LV [28,29]. In the current study, low values of three parameters (GLS, GCS, and GRS) have been independently associated with CHF progression within the next 48 weeks after STEMI.

An integrated approach is essential to apply for assessing the risk of CHF progression. Determination of individual parameters does not reflect a complex clinical and functional picture and cannot be used in patients after STEMI. The best way to assess the risk of developing decompensated CHF is a combination of several parameters reflecting the condition of the cardiovascular system as a whole.

According to the Meta-Analysis Global Group in Chronic Heart Failure (MAGGIC) research, a risk score for prediction of mortality in CHF has been constructed from 13 patient characteristics: age, EF, NYHA class, serum creatinine, diabetes, systolic blood pressure, body mass index, heart failure duration, current smoker, chronic obstructive pulmonary disease, gender, not prescribed a beta-blocker, and not prescribed an ACE inhibitor or ARBs. The difference between the model-predicted and the observed 3-year mortality in the six risk groups varied between 5% and −12% [30]. An addition of BNP or NT-proBNP to the model has significantly increased the predictive significance of the MAGGIC risk score [31].

Other researchers [32] have proposed the BARDICHE-index to assess the risk of hospitalization and mortality in patients with CHF based on body mass index, age, resting systolic blood pressure, dyspnea, NT-proBNP, glomerular filtration rate, resting heart rate, and exercise performance using the six-minute walk test. Outcome has been predicted independently of EF and gender.

As there are no available publications describing the calculation of the risk of CHF progression based on a combined assessment of volumetric and strain characteristics of the LV myocardium together with determination of autonomic rhythm regulation, the approach to risk stratification of STEMI patients is to improve the diagnostic accuracy of prediction. A multivariate diagnostic model for predicting CHF progression within 48 weeks after STEMI has been created and successfully tested. Its high specificity, diagnostic accuracy and sensitivity have been proved in the current study.

## 5. Conclusions

The proposed multivariate model for predicting CHF decompensation within 48 weeks after myocardial infarction, which includes such parameters as heart rate turbulence, left ventricular end-systolic dimension, and global longitudinal strain, has an adequate specificity of 83.3%, a high sensitivity of 95.8%, and a diagnostic accuracy of 93.3%. This technique can be used in the early stages of myocardial infarction to identify patients with a high risk of heart failure progression within the next 48-weeks of postinfarction period.

## 6. Study Limitations

The present study included young and middle-aged patients predominantly (35–65 years old) with primary STEMI with hemodynamically significant stenosis of exclusively infarct-related coronary artery, without a history of CHF. These strict inclusion criteria have been driven by the attempt to exclude the influence of other factors that could lead to myocardial remodeling before the index event (STEMI) and affect the baseline values of the volumetric and deformation characteristics of the myocardium. For this reason, the number of the study participants was relatively small. The authors consider the present study a preliminary one in the process of searching and refining the combination of predictors of the development and progression of CHF after STEMI.

## Figures and Tables

**Table 1:** Parameters correlating with CHF progression within 48 weeks after STEMI.

Variable	Univariate Analysis		Multivariate Analysis	
	RR (95% CI)	*p*	RR (95% CI)	*p*
Pathological TO	2.75 (1.191–6.326)	0.018	-	-
Pathological HRT	2.64 (1.17–5.92)	0.019	3.92 (1.66–9.25)	0.0018
SDANN	0.98 (0.97–1.0)	0.11	-	-
HfP	1.0 (0.99–1.01)	0.22	-	-
BNP	1.001 (1.0001–1.0002)	0.023	-	-
ESD	1.04 (1.01–1.07)	0.0022	1.04 (1.015–1.07)	0.0027
GLS	0.89 (0.82–0.98)	0.017	0.9 (0.815–0.98)	0.028
GCS	0.92 (0.86–0.98)	0.01	-	-
GRS	0.95 (0.92–0.99)	0.012	-	-

Note: BNP—brain natriuretic peptide; HfP—high-frequency component of the spectrum; SDANN—standard deviation of the average values of sinus RR intervals for 5 min; TO—turbulence onset; ESD—end-systolic dimension; RR—relative risk; HRT—heart rate turbulence. The values are given with 95% CI.

**Table 2:** Drug therapy in group “M” and group “T”.

Group of Drugs	Group “M” (*n* = 113)			Group “T” (*n* = 60)			*p* 1–4	*p* 2–5	*p* 3–6
	7th–9th Day	12th Week	48th Week	7th–9th Day	12th Week	48th Week			
	1	2	3	4	5	6			
Beta-blockers	86 (76%)	81 (73%)	74 (65%)	48 (80%)	40 (67%)	36 (60%)	0.56	0.494	0.476
ACE inhibitors/ARBs	93 (82%)	87 (77%)	61 (70%)	53 (88%)	44 (73%)	39 (65%)	0.299	0.594	0.163
Diuretics	21 (19%)	17 (15%)	15 (13%)	9 (15%)	7 (12%)	7 (12%)	0.554	0.541	0.763
Calcium channel blockers	10 (8.8%)	13 (12%)	8 (7.1%)	4 (6.7%)	5 (8.3%)	4 (6.7%)	0.617	0.516	0.919
Class III antiarrhythmic agents	6 (5.3%)	2 (1.8%)	3 (2.7%)	3 (5%)	1 (1.7%)	1 (1.7%)	0.931	0.961	0.681

Note: ARBs—angiotensin II receptor blockers, ACE inhibitors—angiotensin-converting enzyme inhibitors.

**Table 3:** Test results of a model for predicting CHF progression in patients of group “T” (*n* = 60) within 48 weeks after STEMI.

Period after STEMI	12th Week	24th Week	48th Week
True positive, *n*	1	6	10
False positive, *n*	4	2	2
False negative, *n*	2	3	2
True negative, *n*	53	49	46
Sensitivity, %	33.3%	66.7%	83.3%
Specificity, %	93%	96.1%	95.8%
Diagnostic accuracy, %	90%	91.7%	93.3%

## Data Availability

The data presented in this study are available on request from the corresponding author. The data are not publicly available due to ethical reasons.
